# Caffeine Alleviates Oxidative Damage of Retinal Pigment Epithelium Cells

**DOI:** 10.1155/joph/6121757

**Published:** 2025-12-30

**Authors:** Haiyu Liu, Wenwen Zhang, Yucong Xiong, Meiling Yang, Huirong Long, Chaoju Gong, Suyan Li

**Affiliations:** ^1^ Laboratory of Ophthalmology, The Affiliated Xuzhou Municipal Hospital of Xuzhou Medical University, Xuzhou First People’s Hospital, Eye Institute of Xuzhou, Xuzhou, Jiangsu, China; ^2^ Shanghai Key Laboratory of Visual Impairment and Restoration, Shanghai, China, fudan.edu.cn; ^3^ Department of Ophthalmology, The Affiliated Xuzhou Municipal Hospital of Xuzhou Medical University, Xuzhou First People’s Hospital, Eye Institute of Xuzhou, Xuzhou, Jiangsu, China

**Keywords:** apoptosis, ARPE-19, caffeine, NaIO_3_, oxidative damage

## Abstract

**Purpose:**

The purpose of this study was to explore the protective effect of caffeine on oxidative damage of RPE cells and NaIO_3_‐induced retinal degeneration.

**Methods:**

H_2_O_2_ was used to induce the oxidative damage in ARPE‐19 cells. Cell viability was measured by the CCK‐8 assay. The morphology of ARPE‐19 cells was observed by optical microscope. The apoptosis of ARPE‐19 cells was analyzed by Annexin V/PI staining, and DNA fragmentation was detected using the TUNEL assay. The protein levels of apoptosis markers BAX and BCL2 as well as senescence marker p21 were detected by Western blot. DNA damage was indicated by immunofluorescence staining of γ‐H2AX and observed by fluorescence microscopy. Transcriptome profiling of ARPE‐19 cells was performed by RNA‐seq. *In vivo* model of retinal oxidative damage was constructed by injecting NaIO_3_ into the tail vein of C57BL/6 mice. H&E staining was performed after removing the eyeballs.

**Results:**

The CCK‐8 assay showed that caffeine could significantly increase the cell viability inhibited by 200 μM H_2_O_2_. Caffeine significantly reduced H_2_O_2_‐induced DNA fragmentation and apoptosis in ARPE‐19 cells, as demonstrated by the TUNEL assay and Annexin V/PI staining. The results of Western blot showed that caffeine modulated key proteins associated with apoptosis by decreasing BAX and p21 levels while increasing BCL2 expression in H_2_O_2_‐treated ARPE‐19 cells, thereby suggesting its cytoprotective effects. In terms of mechanism, caffeine reduced the levels of reactive oxygen species (ROS) and malondialdehyde (MDA) produced by H_2_O_2_. Caffeine treatment attenuated the accumulation of γ‐H2AX, a marker of DNA damage, in ARPE‐19 cells. Importantly, transcriptome profiling revealed that caffeine might affect complement cascade and lipid metabolism in H_2_O_2_‐treated ARPE‐19 cells. Finally, *in vivo* experiment suggested that chronic administration of caffeine could alleviate oxidative damage of the RPE layer and improve the structure of the entire retina in mice with NaIO_3_‐induced retinal degeneration.

**Conclusion:**

Caffeine can reduce oxidative damage of RPE cells and improve NaIO_3_‐induced retinal degeneration.

## 1. Introduction

Caffeine (1,3,7‐trimethylxanthine), a major component of widely consumed beverages, such as coffee and tea, is one of the most frequently ingested psychoactive substances in the world [1]. Based on data published by the European Food Safety Authority (EFSA), the estimated mean daily caffeine intake among young adults is 37–319 mg [2]. Due to its high lipophilicity and molecular properties, caffeine can easily pass biological barriers. Following ingestion, caffeine is rapidly and almost completely absorbed via the gastrointestinal tract, achieving a bioavailability up to 100% [3]. Caffeine is an adenosine analog that acts as a nonselective antagonist for adenosine receptor (ARs) [4]. While previous studies have demonstrated some evidence that caffeine influences the central nervous system, limited research has explored its effects on the retina. Considering the structural and functional similarities between the brain and the retina, several studies have examined caffeine’s impact on various retinal cell types. In *in vitro* studies using cultured retinal pigment epithelial (RPE) cells, caffeine has been found to inhibit lipopolysaccharide (LPS)‐induced inflammatory response and preserve the integrity of RPE cell layer [5]. Furthermore, caffeine decreases the permeability of RPE cell layer in a hyperglycemic environment through mechanisms involving tight junction repair and apoptosis inhibition [6]. Experimental evidence from both *in vitro* and *in vivo* models indicates that caffeine suppresses choroidal neovascularization by reducing the migratory activity of vascular endothelial cells and inhibiting RPE cell proliferation [7]. Additionally, caffeine consumption has been shown to protect against retinal transient ischemic injury by modulating microglial activation and associated inflammatory response [8]. Despite caffeine’s demonstrated potential for preventing retinal pathologies, the precise molecular and cellular mechanisms underlying these effects remain poorly understood, which hinders the further use of caffeine in patients with retinal diseases.

The retinal pigment epithelium (RPE) is a monolayer between the neural retina and Bruch’s membrane, forming the outer blood–retina barrier (BRB). RPE integrity is critical for retinal functions, such as providing nutrients, maintaining the visual cycle, and phagocytizing photoreceptor membranous disks [9]. As the component of BRB, RPE is vulnerable to the change of systemic homeostasis. The dysfunction and damage of RPE cells is a trigger in the occurrence and development of various retinal diseases, such as age‐related macular degeneration (AMD) and diabetic retinopathy (DR). Intense light exposure, high oxygen partial pressure, and active lipid metabolism jointly promote the formation of highly oxidative environment in RPE cells, which will inevitably lead to oxidative damage. It has been found that the accumulation of oxidative damage is closely related to AMD and DR, leading to protein degeneration, lipid peroxidation, DNA damage, or even RPE death with destruction of BRB [10, 11]. Therefore, it is worth exploring whether caffeine can alleviate oxidative damage of RPE and its mechanisms.

This study aims to explore whether caffeine protects RPE from oxidative stress and its mechanisms, providing experimental evidence for the antioxidative effect of daily caffeine intake on RPE.

## 2. Methods and Materials

### 2.1. Cell Culture

The human retinal pigment epithelial cell line (ARPE‐19) was purchased from the American Type Culture Collection (ATCC, Rockville, MD, USA). The cells were cultured in 1640 culture medium supplemented with 10% fetal bovine serum (Gibco, Waltham, MA, USA) in a humidified cell incubator at 37 °C and 5% CO_2_. Cell passage was performed every two days. Before treatment, the ARPE‐19 cells were seeded at 1 × 10^5^ cells/mL into the 6 cm dish. When cells grew to a density of 70%, the culture medium was replaced with the one containing H_2_O_2_, caffeine, or both for 24 h. Then, cells were collected for further examination. Considering that the half‐life of caffeine in a healthy adult is about 12 h [3], 24‐h treatment of caffeine can take effect on ARPE‐19 cells in a large degree [5, 6], and at this time, the RPE cells were still at the logarithmic phase and kept the favorable biological function.

### 2.2. Cell Viability Assay

The viability of ARPE‐19 cells was assessed by the CCK‐8 reagent (K1018, APExBIO, Houston, USA) according to the manufacturer’s instructions. Firstly, ARPE‐19 cells were seeded onto 96‐well plates at the density of 1 × 10^4^ cells/well. After being cultured for 24 h, the cells were treated with gradient concentrations of caffeine (50, 100, 200, 400, 800, 1000, and 1600 μM) or H_2_O_2_ (25, 50, 100, 200, 400, 800, and 1600 μM) for another 24 h. Secondly, 100 μL of culture medium containing 10 μL of CCK‐8 reagent was added into each well and incubated with cells at 37°C for 4 h. Thirdly, a microplate reader (Synergy H1, BioTek, Winooski, VT, USA) was applied to measure the absorbance (OD) value of each well at a wavelength of 450 nm. The absorbance value of the group without any treatment served as a control. Cell survival rates were calculated using the following formula:

(1) cell survival rate%=absorbance of treated cellsabsorbance of control cells×100%.

### 2.3. TUNEL Assay

The TUNEL assay was used to detect the DNA fragmentation in dying cells. ARPE‐19 cells were cultured directly onto coverslips and fixed with 4% paraformaldehyde at room temperature for 30 min. Then, the cells were permeabilized with 0.2% Triton X‐100 for 5 min and incubated with TUNEL solution (C1086, Beyotime Biotechnology, Shanghai, China) at 37°C in the dark for 1 h. The contour of the cell nucleus was stained by DAPI (1155MG010, BioFroxx, Germany). Finally, cells were visualized and captured using a fluorescence microscope (Axio Observer D1, Zeiss, Oberkochen, Germany). The death rate (%) was calculated based on the following formula:

(2) death rate=number of TUNEL−positive cellsnumber of DAPI nuclei×100%.

### 2.4. Flow Cytometry

Flow cytometry analysis (FCM) was used to assess cell apoptosis after Annexin V‐FITC/PI double staining through the Annexin V‐FITC/PI Apoptosis Kit (K2003, APExBIO). Briefly, ARPE‐19 cells were seeded in 12‐well plates at a density of 1 × 10^5^ cells/well and cultured for 24 h. Then, the cells were treated with 400 μM caffeine or 200 μM H_2_O_2_ for another 24 h. The culture medium was centrifuged to collect floating cells, and the adherent cells were digested using trypsin. All the collected cells were suspended in 200 μL binding buffer containing 5 μL Annexin V‐FITC and 5 μL PI and incubated for 20 min in the dark. The stained cells were detected using a flow cytometer (NL‐CLC, Cytek Biosciences, Shanghai, China) immediately, and the data were analyzed by FlowJo.

### 2.5. Western Blot

ARPE‐19 cells were collected and lysed using RIPA (Millipore, Boston, MA, USA) with 1 mM PMSF and protein phosphatase inhibitor (All‐in‐one, Solarbio, Beijing, China) to extract total proteins. Protein concentration was measured using a Bradford Kit (23238, Thermo Fisher Scientific, Waltham, MA, USA) before denaturing the protein at 100°C for 5 min. Then, the protein samples were subjected to SDS‐PAGE gel electrophoresis. After electrophoresis, the proteins were transferred to nitrocellulose membranes (66485, PALL, Ann Arbor, MI, USA), and the membranes were blocked with 5% nonfat milk at room temperature for 2 h. The membranes were incubated at 4°C overnight with the primary antibodies below: BAX (1 : 1000 dilution, sc‐20067, Santa Cruz, CA, USA), BCL2 (1 : 1000 dilution, 12789‐1‐AP, Proteintech, Rosemont, USA), and p21 (1 : 1000 dilution, 10355‐1‐AP, Proteintech). GAPDH (1 : 1000 dilution, E021010, Earthox, CA, USA) served as a loading control, followed by incubation with the secondary antibody IRDye 800cw goat anti‐mouse IgG (926‐32210, LI‐COR Biosciences, Lincoln, NE, USA, 1 : 10000 dilution) at room temperature for 1 h. Fluorescence signals were visualized and recorded by an Odyssey Infrared Imaging System (LI‐COR Biosciences). The target band was quantified as integrated density using ImageJ software by normalizing to GAPDH.

### 2.6. Measurement of Reactive Oxygen Species (ROS)

The ROS levels in ARPE‐19 cells were determined using 2′,7′‐dichlorodihydroluorescein diacetate (DCFH‐DA) fluorescence probe in the ROS detection kit (S0033S, Beyotime Biotechnology). The DCFH‐DA fluorescence probe was diluted with serum‐free medium to a final concentration of 10 μM. ARPE‐19 cells were seeded onto 6‐well plates at the density of 1 × 10^5^ cells/well and cultured for 24 h. Then, for the caffeine‐treated group, caffeine was added into the culture medium to a final concentration of 400 μM. After 24 h, for the H_2_O_2_‐treated group, H_2_O_2_ was added into the culture medium to a final concentration of 200 μM. 4 h later, the culture medium was discarded, and the cells were incubated with 10 μM DCFH‐DA fluorescence probe solution for 20 min at 37°C in the dark. Finally, the cells were digested using trypsin and put into 96‐well plates. The fluorescence intensity of each well was measured by the microplate reader (Synergy H1) at an excitation wavelength of 488 nm. The fluorescence intensity of each group was normalized to that of the control group.

### 2.7. Measurement of Malondialdehyde (MDA)

The MDA levels in ARPE‐19 cells were determined using thiobarbituric acid (TBA) in the Lipid Peroxidation MDA Assay Kit (S0131S, Beyotime Biotechnology). ARPE‐19 cells were seeded onto 10 cm dishes at the density of 1 × 10^7^ cells/dish. After cultured for 24 h, the cells were treated with 400 μM caffeine or 200 μM H_2_O_2_ for another 24 h. The cells were collected and lysed by RIPA (P0013, Beyotime Biotechnology) and ultrasonic cell crusher (JY96‐IIN, SCIENTZ, Ningbo, China). After centrifugation, the supernatant was mixed with TBA working solution and incubated at 100°C for 15 min. Then, after centrifugation, the supernatant was put into 96‐well plates for measurement. The absorbance (OD) value of each well was measured at 532 nm wavelength with the microplate reader (Synergy H1). The concentration of MDA was calculated according to the standard curve, and the concentration of total protein in each group was quantified by the Bradford assay. The MDA level was indicated by MDA concentration/protein concentration.

### 2.8. Immunofluorescence

To evaluate the degree of DNA damage, immunofluorescence for γ‐H2AX was performed. ARPE‐19 cells were cultured directly onto coverslips and fixed with 4% paraformaldehyde at 4°C for 15 min. After permeabilized with 0.2% Triton X‐100 for 15 min, cells were blocked with 5% bovine serum albumin (9048‐46‐8, Coolaber, Beijing, China) for 2 h at 37°C. Next, the primary antibody of γ‐H2AX (1 : 500 dilution, sc‐517348, Santa Cruz) was used to incubate cells overnight at 4°C, followed by incubation with the fluorescence‐labeled secondary antibody DyLight 488 goat anti‐mouse IgG (H + L) (1 : 500 dilution, E032210, Earthox, CA, USA) for 1 h at 37°C. The contour of the cell nucleus was stained by DAPI (1155MG010, BioFroxx). Finally, cells were visualized and captured using a fluorescence microscope (Axio Observer D1, Zeiss). Integrated density of γ‐H2AX was quantified by ImageJ software.

### 2.9. RNA Sequencing and Analysis

RNA samples were prepared using 1.5 μg of RNA per sample as input. Sequencing libraries were generated using NEBNext®Ultra™RNA Library Prep Kit for Illumina® (NEB, USA) as recommended by the manufacturer, and index codes were added to the attribute sequence for each sample. After the library passed the test, the 5′ end of the library was phosphorylated and looped. The looped library was amplified by rolling loops. Finally, DNA nanospheres (DNB) were loaded into Sequencer and sequenced with DNBSEQ‐T7. The number of reads per gene was counted using HTSeq v0.13.5. FPKM was then calculated for each gene based on its length and the number of reads for that gene. DESeq2 R software package (1.26.0) was used for differential expression analysis between two groups, and KOBAS v3.0 software was used to detect the statistical enrichment of differentially expressed genes (DEGs) in KEGG pathways.

### 2.10. RNA Extraction and Real‐Time Quantitative PCR

ARPE‐19 cells were collected to extract total RNA with RNA‐easy Isolation Reagent (R701, Vazyme, Nanjing, China). The concentration and purity of extracted RNA were determined by OneDrop ultra microphotometer. The All‐in‐one RT SuperMix (R333, Vazyme) was used to reverse‐transcribe RNA to cDNA, and real‐time quantitative PCR was performed using the SYBR Green method. 1 μL cDNA, 4 μL forward and reverse primers (1 μM each), 10 μL SYBR Select Master Mix (Q711, Vazyme), and ddH_2_O were mixed up to a total volume of 20 μL in each reaction tube. The amplification parameters were set as follows: predenaturation at 95°C for 10 min and 40 cycles of amplification (95 °C for 15 s and 60°C for 1 min). The relative levels of mRNA were quantified by the 2^−ΔΔCT^ method with the level of β‐actin as the internal control. The qPCR primer sequences are listed in Table S1.

### 2.11. Retinal Oxidative Damage Mouse Model

The feeding and use of experimental mice were in accordance with the specifications for the use of scientific research animals established by ARVO. The study protocol was reviewed and approved by the Animal Ethics Committee of Xuzhou Medical University (approval number: 202303T030). Twenty‐seven 6‐week‐old male C57BL/6J mice (weight about 20 g) were randomly divided into blank, Caffeine/PBS, Caffeine/NaIO_3_, H_2_O/PBS, and H_2_O/NaIO_3_, with 3 mice in the blank group and 6 mice in each other group. The mice in the Caffeine/PBS and Caffeine/NaIO_3_ groups were fed with 1 g/L concentration of caffeine in drinking water for 4 weeks, and the mice in other groups were fed with normal water. The study involving oral administration of caffeine used concentration (1 g/L) previously reported to have neuroprotective effects in response to CNS injuries and protect retina from transient ischemic damage [8, 12, 13]. At the end of the second week, the model of retinal oxidative damage was established in the Caffeine/NaIO_3_ and H_2_O/NaIO_3_ groups by injecting 20 mg/kg of NaIO_3_ into the tail vein. The mice in the Caffeine/PBS and H_2_O/PBS groups were injected with equivalent PBS through the tail vein, and the mice in the blank group were left untreated. At the end of the 4 weeks, the left eyeball was removed and fixed in FAS eyeball fixative solution (Servicebio, Wuhan, China) under general anesthesia. The mice were sacrificed by excessive anesthesia.

### 2.12. H&E Staining of Mouse Eyeball Sections

The fixed eyeball specimens were routinely embedded in paraffin and serially sectioned along the sagittal plane at a thickness of 5 μm. The slices were routinely deparaffinized and placed in hematoxylin staining solution for 5 min, rinsed with running water and then placed in eosin staining solution, and finally dehydrated and sealed. The slices were observed under an optical microscope (Axio Observer D1, Zeiss). The pictures of retinal sections were captured to analyze the changes of the RPE layer and outer nuclear layer (ONL) thickness.

### 2.13. Statistical Analysis

Statistical data analysis was performed using SPSS v22.0 and GraphPad Prism 5.0. Differences between multiple groups were analyzed by analysis of variance (ANOVA), one‐way ANOVA was performed by Tukey’s or Dunnett’s post hoc test, and two‐way ANOVA was performed by Sidak’s or Tukey’s post hoc test. Data are presented as mean ± SD. *p* < 0.05 was considered statistically significant.

## 3. Results

### 3.1. Caffeine Protects ARPE‐19 Cells From H_2_O_2_‐Induced Oxidative Stress

To investigate the effects of varying concentrations of caffeine on ARPE‐19 cells, the indicated gradient concentrations of caffeine were used to treat ARPE‐19 cells for 24 h. The CCK‐8 assay revealed that concentrations of caffeine ≥ 800 μM had a direct detrimental effect on cell viability (Figure 1(a)). Therefore, in consideration of the caffeine concentrations used in previous studies [5, 7], 400 μM caffeine was selected for further experiments to minimize potential cytotoxicity to ARPE‐19 cells. Then, cell viability of ARPE‐19 cells with or without caffeine (400 μM) treatment was examined after adding the indicated gradient concentrations of H_2_O_2_ for 24 h. The results of the CCK‐8 assay demonstrated that caffeine treatment significantly increased the cell viability suppressed by 100 μM and 200 μM H_2_O_2_ (Figure 1(b)). Consistent with these findings, cell morphology confirmed that caffeine protected ARPE‐19 cells from the oxidative damage induced by H_2_O_2_ (Figure 1(c)). Collectively, the above findings reveal that caffeine exerts antioxidative effects in ARPE‐19 cells.

Figure 1Caffeine protects ARPE‐19 cells from H_2_O_2_‐induced oxidative stress. (a) ARPE‐19 cells were treated with different concentrations of caffeine for 24 h, and the cell survival rate was measured using CCK8. The statistical analysis was performed by one‐way ANOVA with Dunnett’s post hoc test (*n* = 6). (b) ARPE‐19 cells were incubated with various concentrations of H_2_O_2_ and caffeine (400 μM) simultaneously for 24 h, and the cell survival rate was measured using CCK8. The statistical analysis was performed by two‐way ANOVA with Sidak’s post hoc test (*n* = 3). (c) The morphological changes in ARPE‐19 cells following indicated treatments. Black arrows indicated contracted and aggregated dying cells.

**Figure:**
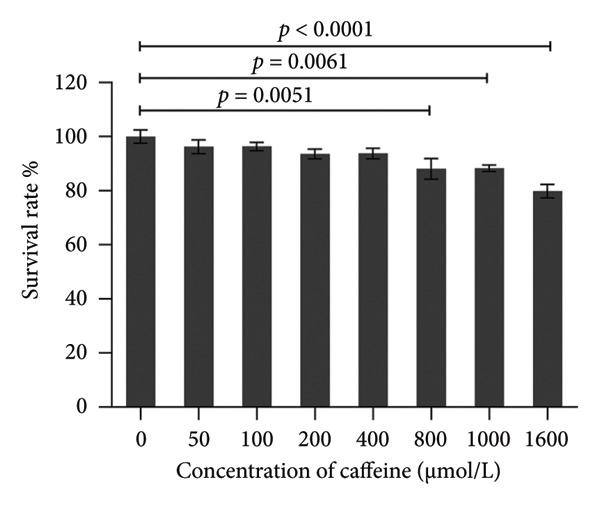
(a)

**Figure:**
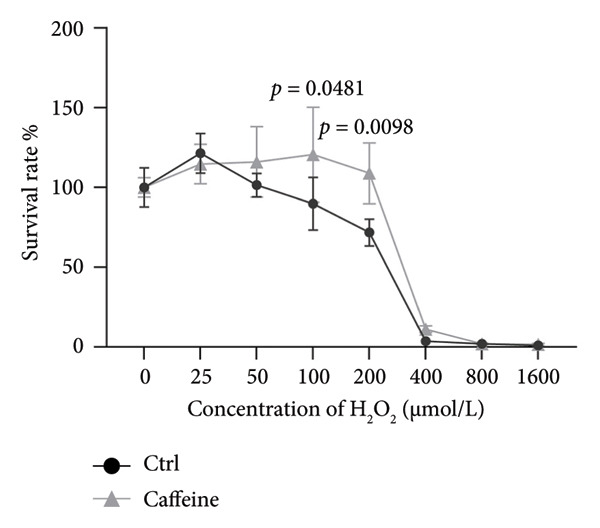
(b)

**Figure:**
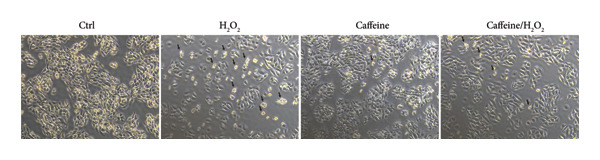
(c)

### 3.2. Caffeine Inhibits the Apoptosis of ARPE‐19 Cells Treated With H_2_O_2_

To investigate whether caffeine could alleviate the H_2_O_2_‐induced apoptosis, the apoptosis assays were conducted in the ARPE‐19 cells exposed to H_2_O_2_ with or without caffeine treatment. TUNEL staining revealed that the percentage of TUNEL‐positive cells increased to 26.3% after treating with H_2_O_2_ while that was evidently decreased to 2.8% in the presence of caffeine (400 μM) (Figure 2(a)). FCM indicated that the percentage of early and late apoptotic cells rose to 14.8% after adding H_2_O_2_, and caffeine reduced the percentage to 3.4% (Figure 2(b)). When studying apoptosis‐related markers, it was found that the expression ratio (BAX/BCL2) was upregulated by H_2_O_2_ but downregulated by caffeine (Figure 2(c)), suggesting that caffeine antagonized the apoptosis of ARPE‐19 cells through canonical apoptosis pathway. In addition, H_2_O_2_ induced the expression of p21, a cell cycle protein involving apoptosis, which was inhibited by caffeine (Figure 2(c)). Therefore, caffeine can reverse the H_2_O_2_‐induced apoptosis of ARPE‐19 cells to some extent.

Figure 2Caffeine inhibits the apoptosis of ARPE‐19 cells treated with H_2_O_2_. (a) The DNA fragmentation after the indicated treatments for 24 h was shown using TUNEL staining. The statistical analysis was performed by one‐way ANOVA with Tukey’s post hoc test (*n* = 3). (b) The apoptosis rates of ARPE‐19 cells after the indicated treatments for 24 h were quantified by Annexin V/PI staining and flow cytometry. The statistical analysis was performed by one‐way ANOVA with Tukey’s post hoc test (*n* = 3). (c) Western blot analysis of BCL2, BAX, and p21 protein levels in ARPE‐19 cells after the indicated treatments for 24 h. The ratios of BAX/BCL2 and p21/GAPDH were calculated based on the band density quantified by ImageJ. The statistical analysis was performed by one‐way ANOVA with Tukey’s post hoc test (*n* = 3).

**Figure:**
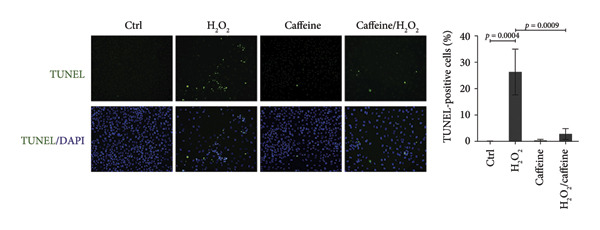
(a)

**Figure:**
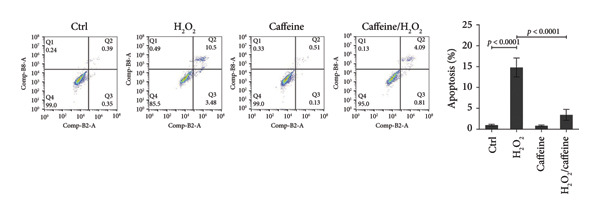
(b)

**Figure:**
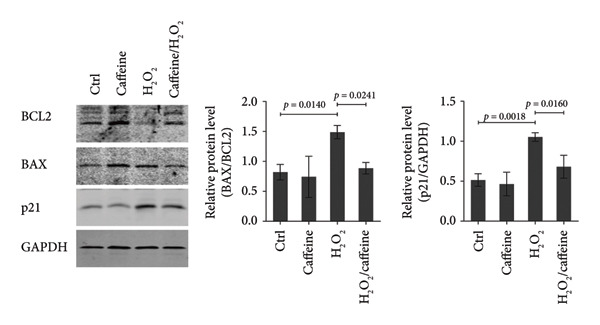
(c)

### 3.3. Caffeine Alleviates H_2_O_2_‐Induced Oxidative Damage of ARPE‐19 Cells

To investigate the antioxidative capacity of caffeine in ARPE‐19 cells, the levels of ROS and MDA, an indicator of membrane lipid peroxidation, were assessed in the ARPE‐19 cells treated and exposed to H_2_O_2_ with or without caffeine treatment. As shown in Figure 3(a), the relative level (normalized to the control group) of ROS was elevated by H_2_O_2_ treatment, and caffeine treatment inhibited the increase of ROS. Compared with the control group, the level of the lipid peroxidation product MDA exhibited a 3.5‐fold increase following H_2_O_2_ treatment. However, caffeine treatment significantly suppressed this increase in MDA levels (Figure 3(b)). Furthermore, immunofluorescence analysis was used to evaluate the expression level and localization of DNA damage marker γ‐H2AX in ARPE‐19 cells. The results showed that H_2_O_2_ treatment led to a marked increase in the nuclear accumulation and fluorescence intensity of γ‐H2AX staining compared to the control group, while the increased intensity of γ‐H2AX staining was attenuated by caffeine treatment (Figure 3(c)). The above findings suggest that caffeine exerts the antioxidative effect on ARPE‐19 cells.

Figure 3Caffeine alleviates H_2_O_2_‐induced oxidative damage of ARPE‐19 cells. (a) ROS in ARPE‐19 cells with the indicated treatments was measured using ROS detection probe as indicator and quantified by fluorescence intensity in microplate reader. The statistical analysis was performed by one‐way ANOVA with Tukey’s post hoc test (*n* = 3). (b) MDA in ARPE‐19 cells with the indicated treatments for 24 h was measured using the TBA method and quantified by absorbance in microplate reader. The statistical analysis was performed by one‐way ANOVA with Tukey’s post hoc test (*n* = 3). (c) Immunofluorescence showed the expression level and localization of DNA damage marker γ‐H2AX in ARPE‐19 cells after the indicated treatments for 24 h. Integrated density of γ‐H2AX/nucleus numbers was quantified by ImageJ. The statistical analysis was performed by one‐way ANOVA with Tukey’s post hoc test (*n* = 3).

**Figure:**
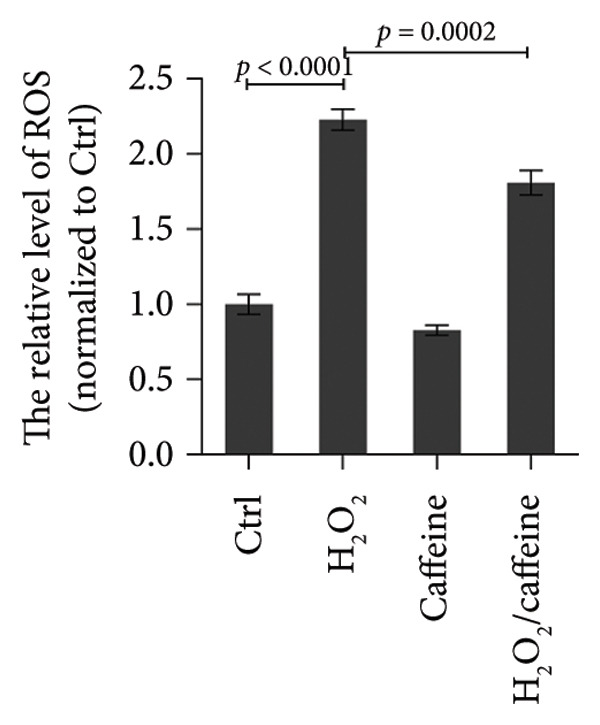
(a)

**Figure:**
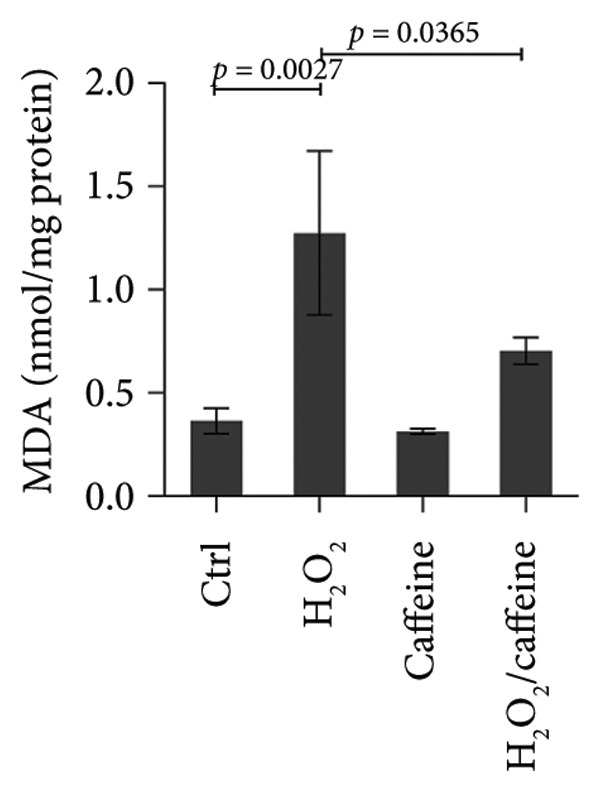
(b)

**Figure:**
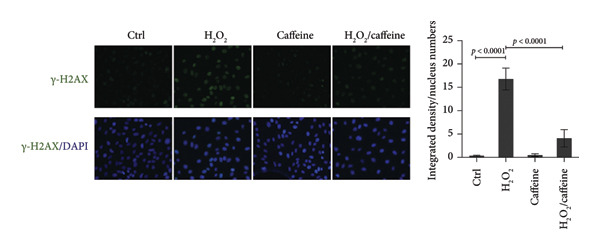
(c)

### 3.4. Caffeine Represses the Pathways of Complement Cascade and Lipid Metabolism in ARPE‐19 Cells Treated With H_2_O_2_

To further elucidate the protective mechanisms of caffeine in RPE cells, the transcriptomic differences between ARPE‐19 cells with or without H_2_O_2_ and caffeine treatment were analyzed using RNA‐seq technology. The differential expression analysis revealed 171 upregulated genes and 194 downregulated genes in caffeine and H_2_O_2_‐cotreated ARPE‐19 cells, compared with H_2_O_2_‐treated ARPE‐19 cells (Table S2). A pathway enrichment analysis on DEGs was conducted to investigate their functional roles. It was found that the enrichment pathway of downregulated genes following caffeine treatment included complement cascade and lipid metabolism which might be associated with H_2_O_2_‐induced oxidative damage and cell death of ARPE‐19 (Figure 4(a)). Furthermore, the DEGs categorized into complement cascade and lipid metabolism pathways were validated using qPCR analysis. For complement cascade, the results showed that C3AR1, C1QB, and C1QC mRNAs were induced by H_2_O_2_ but were decreased when adding caffeine. For lipid metabolism, H_2_O_2_‐induced upregulation of PLA2G2A and PLA2G4F mRNAs was inhibited by caffeine (Figure 4(b)). These data suggest that caffeine may repress the activation of complement cascade and lipid metabolism in ARPE‐19 cells treated with H_2_O_2_.

Figure 4Caffeine represses the pathways of complement cascade and lipid metabolism in ARPE‐19 cells treated with H_2_O_2_. (a) The transcriptome differences between ARPE‐19 cells with or without H_2_O_2_ and caffeine treatments for 24 h were analyzed by RNA‐seq. KEGG pathway enrichment analysis showing the upregulated and downregulated genes in the indicated comparison. (b) The qPCR analysis of *C1QB*, *C1QC*, *C3AR1*, *PLA2G2A*, and *PLA2G4F* mRNA levels in ARPE‐19 cells with the indicated treatments for 24 h. The statistical analysis was performed by one‐way ANOVA with Tukey’s post hoc test (*n* = 3).

**Figure:**
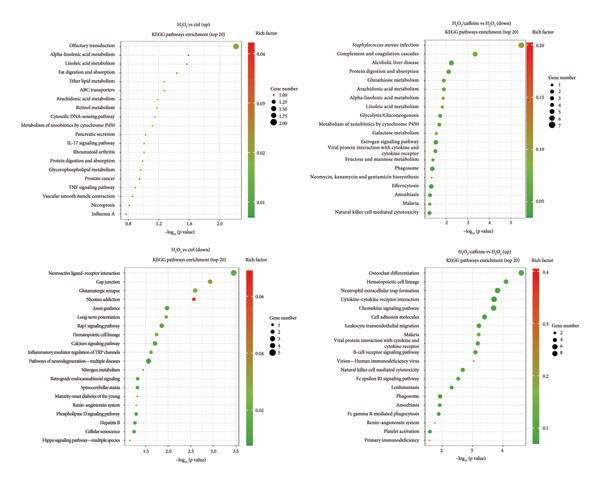
(a)

**Figure:**
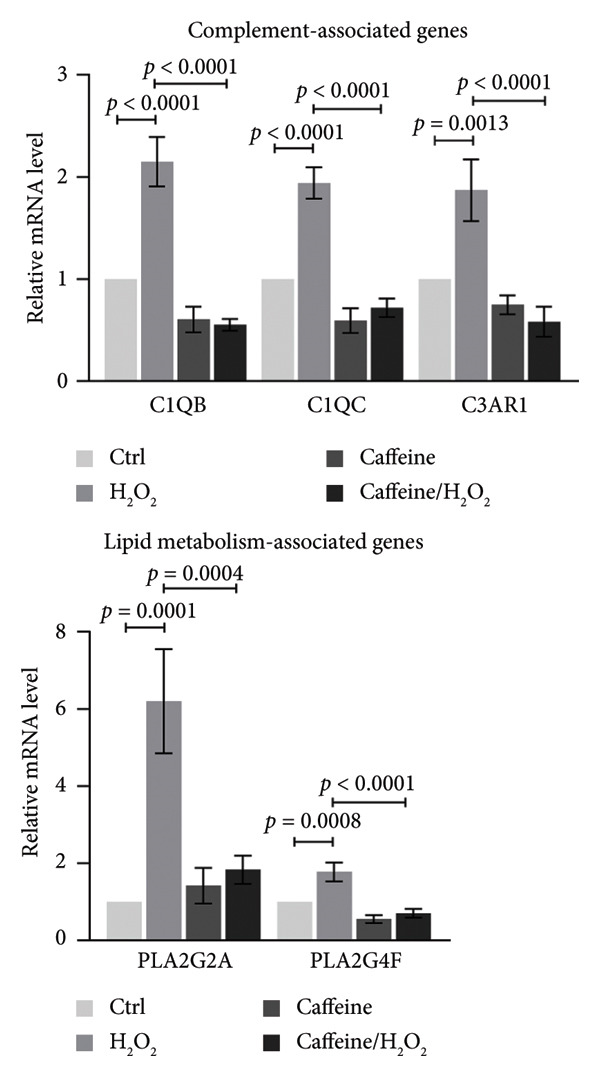
(b)

### 3.5. Caffeine Reduces NaIO_3_‐Induced Oxidative Damage of RPE Cells *In Vivo*

To mimic the effects of long‐term caffeine consumption *in vivo*, caffeine‐containing drinking water was administrated via drinking water for 4 weeks, after which NaIO_3_ was injected intravenously into the tail vein (Figure 5(a)). H&E staining of retinal sections from H_2_O/NaIO_3_‐treated mice indicated depigmentation and swelling of the RPE layer, accompanied by disorganization and thinning of ONL. Notably, pronounced swelling was observed in the outer plexiform layer. Importantly, long‐term caffeine consumption markedly attenuated the above abnormalities in the retinas of NaIO_3_‐treated mice (Figure 5(b)). In addition, no retinal abnormalities were observed in the Caffeine/PBS group compared with the blank and H_2_O/PBS groups, suggesting that long‐term oral administration of 1 g/L caffeine does not induce retinal damage and is therefore protective for mice under these conditions. In summary, long‐term intake of caffeine may alleviate oxidative damage of RPE cells and improve the structure of the entire retina.

Figure 5Caffeine reduces NaIO_3_‐induced oxidative damage of RPE cells *in vivo*. (a) Schematic diagram showing the procedures of tail intravenous injection of NaIO_3_ and caffeine feeding. (b) The representative images of H&E staining of the retinal sections around 500 μm from the optic nerve head in the indicated groups. Thickness of the outer nuclear layer (ONL) in the indicated groups (*n* = 6). ^∗^ *p* versus H_2_O/PBS; ^#^ *p* versus H_2_O/NaIO_3._ The statistical analysis was performed by two‐way ANOVA with Tukey’s post hoc test (*n* = 6).

**Figure:**
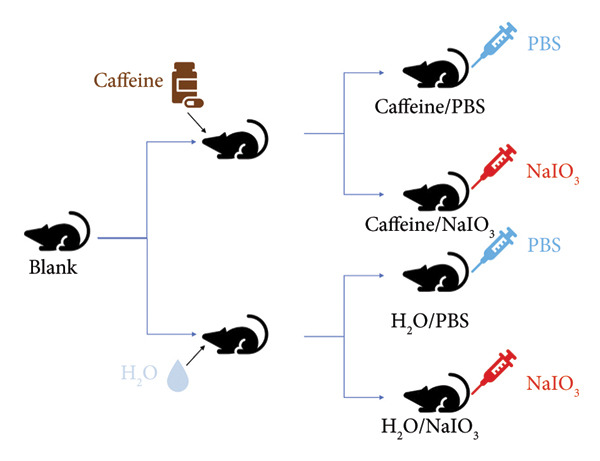
(a)

**Figure:**
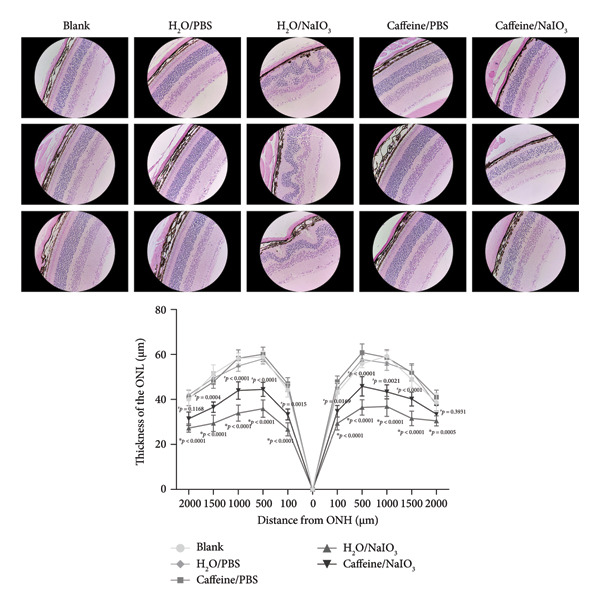
(b)

## 4. Discussion

Caffeine bonds with ARs, including A1R, A2AR, A2BR, and A3R, acting as a competitive antagonist of adenosine. Although caffeine interacts with all AR subtypes, its strongest binding affinities are observed for A1R and A2AR [14]. A1R and A2R are widely expressed in the retina. A1R and A2BR are primarily expressed in the choroid and RPE, with higher abundance in the neural retina. On the contrary, the expression of A2AR is relatively low in the neural retina, with higher levels observed in the choroid and RPE [15]. Based on these findings, it was hypothesized that caffeine exerts a significant influence on the RPE. Notably, previous research has primarily concentrated on the effects of caffeine on the microglia and vascular endothelial cell in the retina. In the models of glaucoma and ischemia reperfusion, caffeine significantly suppressed the activation of retinal microglia, thereby providing neuroprotective effects [8, 16]. The migration of retinal endothelial cells serves as a critical step in the process of neovascularization. It was found that the migration of choroidal and retinal vascular endothelial cells was suppressed after caffeine treatment [7]. In addition, caffeine directly prevented pathological neovascularization in oxygen‐induced retinopathy by reducing the proliferation of vascular endothelial cells [17]. In this study, it was found that caffeine could decrease the apoptosis of RPE cells under oxidative stress. Further investigations showed that intracellular ROS and lipid peroxidation product MDA in the RPE cells were reduced by caffeine, which was consistent with the previous reports revealing the antioxidant function of caffeine [18, 19]. More importantly, the DNA oxidative damage of RPE cells was relieved after caffeine treatment. All the above findings suggest that caffeine may protect RPE cells from oxidative stress by scavenging ROS.

Although caffeine exhibits antioxidant properties, its molecular mechanisms remain unclear. In addition to its role as an AR antagonist, caffeine also acts as a nonselective inhibitor of phosphodiesterase (PDE) involved in the degradation of cyclic adenosine monophosphate (cAMP) to the noncyclic form 5′‐AMP [20]. Through this mechanism, caffeine elevates the intracellular cAMP level, which subsequently upregulates the level of glutathione (GSH) and the activity of mitochondrial SOD2 through activating protein kinase A (PKA). This results in the neutralization of ROS [21]. Moreover, caffeine promoted the expressions of nuclear factor erythroid 2‐related factor 2 (NRF‐2) and downstream heme oxygenase‐1 (HO‐1) for antagonizing oxidative stress [22]. To further explore the protective mechanisms of caffeine in RPE cells, the changes in the transcriptomes of RPE cells after caffeine treatment were analyzed. It was noticed that caffeine downregulated several genes involved in two pathways, complement cascades and lipid metabolism pathways. In RPE cells, complement pathway activation always occurred simultaneously with oxidative stress and forms a channel that penetrates the cell membrane, leading to cell death [23]. It had been found that oxidative stress not only dysregulated endogenous complements of RPE cells but also rendered RPE cells susceptible to complement‐mediated injury [24, 25]. Notably, interfering with the alternative complement pathway protected RPE cells from oxidative stress [26]. Here, it was found that oxidative stress induced the expressions of complement‐associated genes C1QB, C1QC, and C3AR1 in RPE cells, which were suppressed by caffeine, suggesting that caffeine may protect RPE cells from oxidative stress through inhibiting the activation of complement pathway. Except for complements, it was also found that PLA2G2A and PLA2G4F, two members of phospholipase A2 (PLA2) family, were reduced by caffeine in the RPE cells with oxidative stress. PLA2 enzymes cleave fatty acids at the sn‐2 position of glycerol phospholipids to give free fatty acid and lysophospholipid. Released lipid acids were further oxidatively metabolized, producing various free radical intermediates along with the production of hydroxyl and superoxide radicals [27]. Therefore, high expression of PLA2 was closely related to oxidative stress [28, 29]. In combination with the previous studies, the findings indicate a possibility that caffeine reduces the generation of ROS by regulating lipid metabolism.

To investigate the protective effect of caffeine on RPE *in vivo*, oral administration of caffeine was adopted in the mice with retinal oxidative damage. The mice were provided with the water containing caffeine (1 g/L) every day, resulting in a plasma concentration of 50 μM, equivalent to the level achieved in humans by consuming approximately five cups of coffee a day [12]. Following intravenous injection of NaIO_3_ to induce a model of retinal oxidative damage, caffeine was administrated to the mice for an additional 2 weeks to assess the long‐term protective effects of caffeine. Long‐term oral administration of caffeine was nontoxic to the retina but could obviously improve the overall retinal structure in mice with oxidative damage, suggesting that caffeine may exert a protective role in multiple retinal cell types, including but not limited to RPE cells.

There are some limitations in this study. For example, the effects of caffeine are different depending on the dose used for the antagonism of ARs and the inhibition of PDE enzymes [2]; however, the antioxidant capabilities of caffeine at different doses were not explored. Next, the mechanisms of caffeine against oxidative stress in RPE had not been deeply studied. Finally, considering that coffee contains a variety of active ingredients and that the composition of each active ingredient in different types of coffee is different, as a single component, caffeine cannot fully explain the benefits of daily coffee consumption. The roles of other components in coffee should also be explored.

Taken together, the findings of this study demonstrate that caffeine can protect RPE cells from oxidative stress *in vitro* and improve the NaIO_3_‐induced oxidative damage of mouse retina, thus providing experimental evidence for the benefit of daily coffee on the retina.

## Ethics Statement

The protocol of the animal experiment was reviewed and approved by the Animal Ethics Committee of Xuzhou Medical University (approval number: 202303T030).

## Disclosure

All authors approved the final manuscript.

## Conflicts of Interest

The authors declare no conflicts of interest.

## Author Contributions

Haiyu Liu, Wenwen Zhang, Yucong Xiong, Chaoju Gong, and Suyan Li provided the conception and designed the experiments. Haiyu Liu, Wenwen Zhang, Yucong Xiong, Meiling Yang, Huirong Long, and Chaoju Gong performed the experiments. Haiyu Liu, Yucong Xiong, and Chaoju Gong analyzed and interpreted data. Haiyu Liu, Wenwen Zhang, Yucong Xiong, and Chaoju Gong drafted the manuscript. Haiyu Liu, Wenwen Zhang, Yucong Xiong, Chaoju Gong, and Suyan Li revised the manuscript. Chaoju Gong and Suyan Li acquired the funding. Haiyu Liu, Wenwen Zhang, and Yucong Xiong contribute equally to the manuscript.

## Funding

This study was supported by the Natural Science Foundation of Jiangsu Province (BK20211052), Jiangsu Training Program of Innovation and Entrepreneurship for Undergraduates (202210313031Z), Basic Research Plan of Xuzhou Science and Technology Project (KC23071), and Open Projects of Key Laboratories in Colleges and Universities in Jiangsu Province (XZSYSKF2022010).

## Supporting Information

Additional supporting information can be found online in the Supporting Information section.

## Supporting information


**Supporting Information 1** Table S1: The sequences of qPCR primers.

**Supporting Information 2** Table S2: The differentially expressed genes in the comparison of H_2_O_2_ vs. Ctrl.

**Supporting Information 3** Table S3: The differentially expressed genes in the comparison of H_2_O_2_/Caffeine vs. H_2_O_2_.

## Data Availability

The datasets used and/or analyzed during this study are available from the corresponding author upon reasonable request.
